# Corticosterone induces obesity partly *via* promoting intestinal cell proliferation and survival

**DOI:** 10.3389/fendo.2022.1052487

**Published:** 2023-01-09

**Authors:** Guanhao Wang, Shuanqing Li, Yingqi Li, Meihui Zhang, Ting Xu, Tianming Li, Lining Cao, Jianfeng Lu

**Affiliations:** ^1^Shanghai YangZhi Rehabilitation Hospital (Shanghai Sunshine Rehabilitation Center), Frontier Science Center for Stem Cell Research, School of Life Sciences and Technology, Tongji University, Shanghai, China; ^2^Institute of Biophysics, Chinese Academy of Sciences, Beijing, China; ^3^College of Life Sciences, University of Chinese Academy of Sciences, Beijing, China; ^4^Biomedical research center, Suzhou Institute of Tongji University, Suzhou, China

**Keywords:** corticosterone, obesity, small intestines, cell proliferation, cell survival and death

## Abstract

**Introduction:**

A vicious cycle ensues whereby prolonged exposure to social stress causes increased production of glucocorticoids (GCs), leading to obesity even further. Understanding the role of GCs, the key element in the vicious circle, might be helpful to break the vicious circle. However, the mechanism by which GCs induce obesity remains elusive.

**Methods:**

Corticosterone (CORT) was administered to mice for 8 weeks. Food and water intake were recorded; obesity was analyzed by body-weight evaluation and magnetic resonance imaging (MRI); intestinal proliferation and survival were evaluated by H&E staining, EdU-progression test, TUNEL assay and immunofluorescence staining of Ki67 and CC3; RNA-seq was performed to analyze transcriptional alterations in small intestines and livers.

**Results:**

Chronic CORT treatment induced obesity, longer small intestines, hepatic steatosis and elevated levels of serum insulin and leptin in mice; CORT-treated mice showed increased cell proliferation and decreased apoptosis of small intestines; RNA-seq results indicate that differentially expressed genes (DEGs) were enriched in several cell growth/death-associated signaling pathways.

**Discussion:**

Herein we find that administration of CORT to mice promotes the proliferation and survival of intestinal cells, which might contribute to the longer small intestines and the elongated intestinal villi, thus leading to increased nutrient absorption and obesity in mice. Understanding CORT-induced alterations in intestines and associated signaling pathways might provide novel therapeutic clues for GCs or stress-associated obesity.

## 1 Introduction

Obesity, which threatens the health of individuals, is a rapidly increasing pandemic. It is predicted that one fifth of adults worldwide will be expected to be obese by 2025, and the economic cost for chronic diseases due to obesity is estimated to be 990 billion US dollars per year globally (1). In the past few decades, excessive food intake or insufficient energy expenditure was considered to contribute to obesity. However, recent evidence is now mounting to suggest that stress-induced glucocorticoids (GCs) elevations play an important role in obesity (2, 3).

Accumulated evidence suggested that stress plays an important role in the development of obesity. Hypothalamic-pituitary-adrenocortical (HPA) axis, a well-known neuroendocrine system mediating stress response, stimulates the adrenal cortex to release GCs: cortisol in humans or corticosterone (CORT) in rodents into blood. Patients chronically exposed to high levels of GCs, like in Cushing’s syndrome (endogenous GCs) or when using high doses of exogenous GCs, are more likely to develop abdominal obesity (4, 5). Interestingly, in our modern society, the obesity pandemic coincides with an increase of factors that enhance GCs production, such as chronic stress, consumption of food with a high glycemic index, and a reduced amount of sleep. This thus indicated a vicious circle, within which the increased GCs action, obesity and stress interact and intensify with each other (6), leading inexorably to worsening of obesity and metabolic status. Understanding the role of GCs, the key element in the vicious circle, might be helpful to break the vicious circle. However, the mechanism by which GCs induce obesity is largely unknown.

Excessive food intake and nutrient absorption are likely to contribute to obesity. Small intestine is one of the major organs for nutrient absorption. Intestinal villi are tiny, finger-like structures which project into the intestinal cavity, greatly increasing the surface area for nutrients absorption. The remarkable length of the small intestines, determined by a balance between the rates of proliferation and death of intestinal cells, contributes greatly to its surface area for nutrient absorption (7–9). A recent study indicated that CORT-treated animals became obese, even when the food intake was restricted to the same amount as consumed by the control mice (10). This gives rise to the question whether GCs could regulate proliferation and survival of intestinal cells, thus leading to an increased gut absorptive capacity, which results in obesity.

In the present study, CORT was administered to mice, and the small intestines and the livers were evaluated. We found that CORT-treated mice displayed an obese phenotype along with longer small intestines and elongated intestinal villi, which might be the result of increased proliferation and decreased death of intestinal cells. RNA-seq analysis suggested that CORT-treatment significantly increased the expression levels of proliferation-related genes (such as *Glul*, *Wee1* and *Xiap*) and decreased the expression levels of apoptosis-related genes (such as *Casp1* and *Casp7*) in small intestines. Furthermore, in the liver, the expression levels of *Fgf1* and *Fgf21*, which are known to regulate the length of intestines, were also up-regulated. We concluded that CORT induced longer small intestines and villi *via* promoting intestinal cell proliferation and survival, which contributed to obesity. This study would provide a novel therapeutic clue for the stress/GCs-related obesity.

## 2 Result

### 2.1 Chronic CORT treatment induced obesity, longer small intestines, hepatic steatosis and elevated levels of serum insulin and leptin in mice

**Figure 1:**
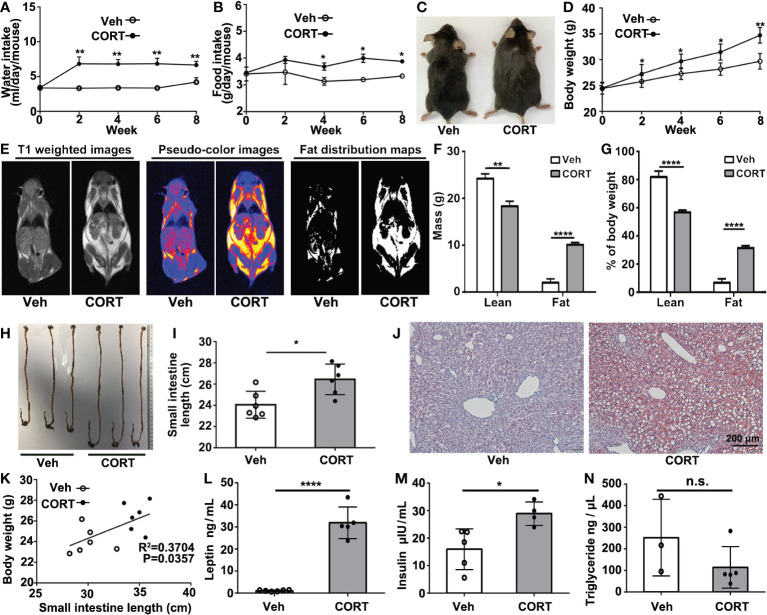
CORT induced obese phenotype, increased intestinal length, hepatic steatosis and changes in serum. **(A)** Water consumption and **(B)** food consumption of mice injected with CORT or Vehicle. **(C)** Representative images comparing the body size of a CORT-treated mouse with a vehicle-control mouse. **(D)** Body weight curves of mice treated with CORT or vehicle for 8 weeks. **(E)** Representative MRI images for body composition analysis. **(F)** Evaluation of fat mass. **(G)** Evaluation of fat percentage. **(H)** The representative images of the intestines of the mice treated with CORT or vehicle. **(I)** Quantification of the length of the small intestines from the mice treated with CORT or vehicle. **(J)** Representative images of Oil red O staining of the liver sections. **(K)** Correlation analysis between body weight and small intestine length. **(L)** Detection of the concentrations of serum leptin, **(M)** insulin and **(N)** triglyceride for the two groups of mice. Data are expressed as mean ± SEM, *p < 0.05, **p < 0.01, ****p < 0.0001. Veh: vehicle-treated group; CORT, corticosterone-treated group; n.s., not significant.

After 8 weeks of treatment with CORT, mice showed remarkable increases in water intake, food intake, body weight and body size (**Figures 1A–D**). Magnetic resonance imaging (MRI) revealed that the CORT-treated mice showed remarkable fat accumulation (**Figure 1E**). According to the T1-weighted images, pseudo-color images, and fat distribution maps, CORT-treated mice showed a higher fat mass (10.10 ± 0.26 *vs*. 1.98 ± 0.50 g, p=0.0001) and a higher percentage of fat (31.37 ± 1.00 *vs*. 6.66 ± 1.69%, p = 0.0002) (**Figures 1F, G**). Histological analysis showed longer small intestines (**Figures 1H, I**) and hepatic steatosis (**Figure 1J**). Furthermore, we found a positive relationship between the body weight and the length of small intestines (**Figure 1K**). Chronic CORT exposure led to significantly elevated levels of serum leptin (31.91 ± 3.21 *vs*. 1.08 ± 0.12 ng/mL, p<0.0001) and insulin (28.90 ± 2.15 *vs*. 15.97 ± 3.31 μIU/mL, p=0.0179) (**Figures 1L, M**); however, there is no significant difference in the level of serum triglyceride (114.30 ± 43.00 *vs*. 252.10 ± 102.30 ng/μL, p=0.1939) between the two groups (**Figure 1N**). This result indicated that CORT-treatment increased the length of small intestines, which might contribute to more nutrients absorption, fat accumulation and weight gain.

### 2.2 CORT-treated mice showed increased cell proliferation and decreased apoptosis of small intestines

**Figure 2:**
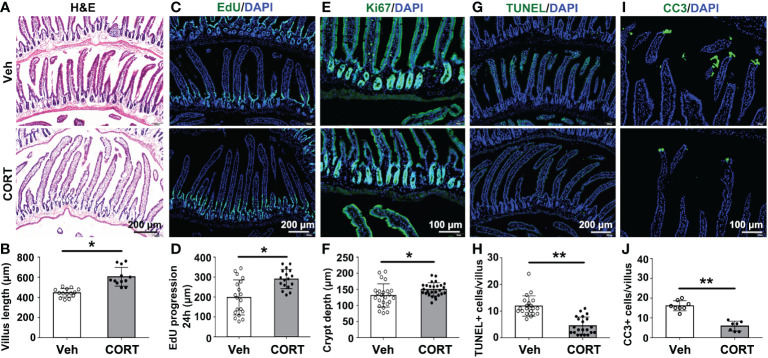
CORT increased intestinal villus length by breaking the balance between proliferation and death of intestinal cells. **(A)** Hematoxylin and eosin (H&E)-staining for duodenum sections. **(B)** Quantitative analysis of villi length for duodenum from Vehicle- or CORT-treated mice. **(C)** Immunofluorescence staining of EdU for the duodenum sections from Vehicle- or CORT-treated mice. **(D)** Quantification of the progression of EdU-labelled cells along the duodenum villi from Vehicle- or CORT-treated mice. **(E)** Immunofluorescence staining of Ki67 for the duodenum sections from Vehicle- or CORT-treated mice. **(F)** Quantification of the duodenal crypt depth indicated by Ki67-staining from Vehicle- or CORT-treated mice. **(G)** Immunofluorescence staining of TUNEL for the duodenum sections from Vehicle- or CORT-treated mice. **(H)** Quantification of TUNEL-positive cells of duodenal crypt from Vehicle- or CORT-treated mice. **(I)** Immunofluorescence staining of CC3 for the duodenum sections from Vehicle- or CORT-treated mice. **(J)** Quantification of CC3-positive cells of duodenal crypt from Vehicle- or CORT-treated mice. Data are expressed as mean ± SEM, *p < 0.05; **p < 0.01. CC3: Cleaved Caspase 3. Veh: vehicle-treated group; CORT: corticosterone-treated group.

The tiny finger-like intestinal villi in the small intestines, projecting into the intestinal cavity, greatly increase the intestinal surface area for nutrients absorption. Several studies implied that the increase in villi length might contribute to increased nutrient absorption, weight gain and fat accumulation (7–9). In the present study, CORT-treated mice showed longer intestinal villi (605.10 ± 26.89 μm, n=12) than that in the control mice (444.80 ± 11.24 μm, n=14) (**Figures 2A, B**). The length of intestinal villus is determined by the balance between the rates of proliferation and death of intestinal cells, so we then evaluated cell proliferation and apoptosis in intestinal villi. EdU (ethynyl-2’-deoxyuridine), which could incorporate into newly synthesized DNA of dividing cells during the S phase (11), was administered by intraperitoneal injection to quantify intestinal cell proliferation and migration as previously described (12). In CORT-treated mice, small intestines showed remarkable increase in EdU progression (**Figures 2C, D**) and deeper crypt depth (**Figures 2E, F**; labeled with Ki67 and measured as the distance from the crypt mouth to the crypt base), indicating an increased proliferation rate. Furthermore, CORT-treated mice showed fewer TUNEL-positive (**Figures 2G, H**) or Cleaved Caspase 3 (CC3)-positive cells (**Figures 2I, J**) at the villus tip, indicating delayed shedding at the tip and decreased intestinal cell death by apoptosis. This result indicated that CORT-treatment regulated intestinal cell proliferation and survival.

### 2.3 Identification of differentially expressed genes in response to the CORT treatment in small intestines

**Figure 3:**
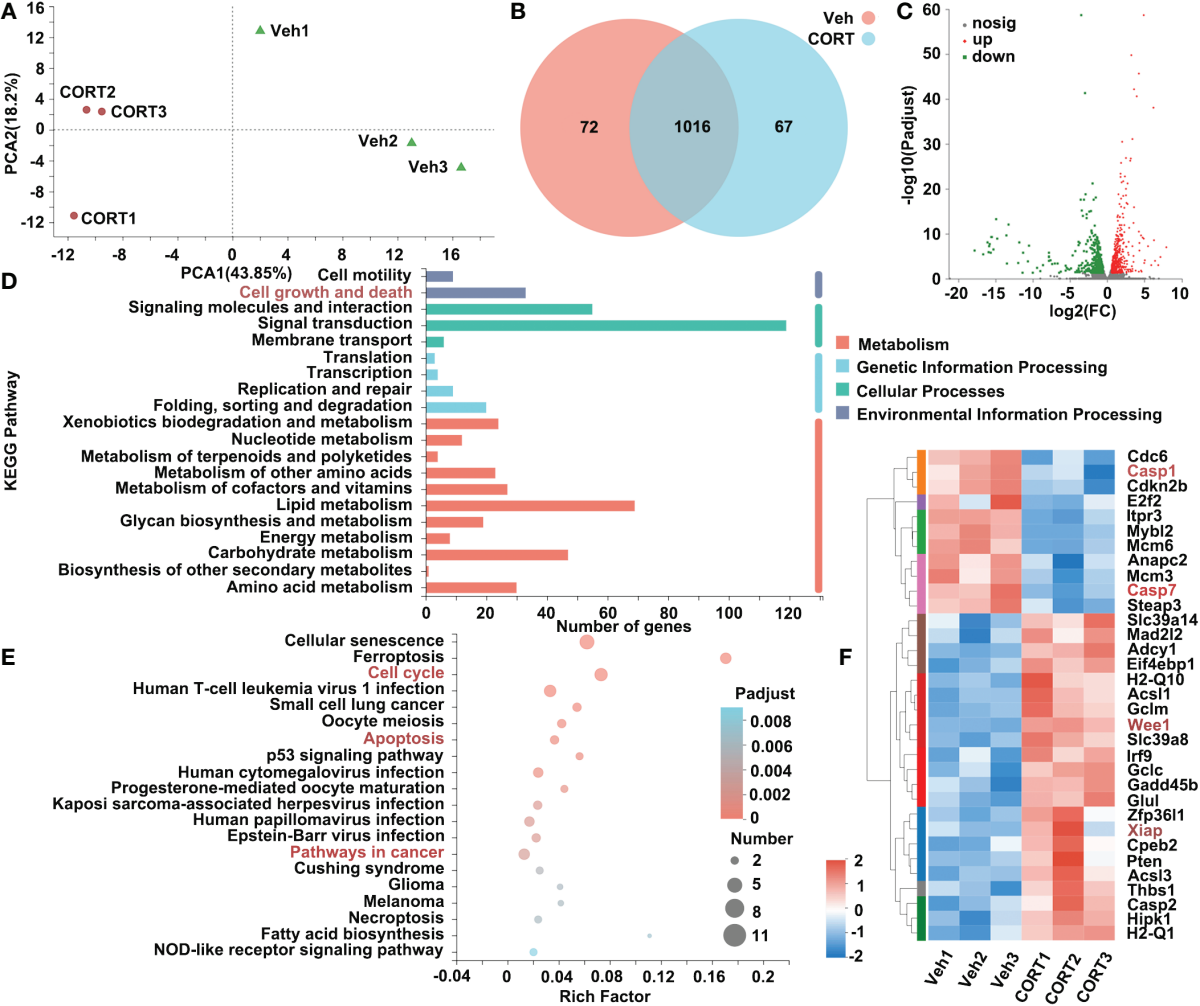
Identification of genes that were differentially expressed in intestines in response to CORT treatment. **(A)** Principal component analysis of the transcriptome of the intestine samples collected from the mice administrated with CORT or Vehicle. **(B)** Venn diagram displaying the distribution of DEGs (genes with > 1.5-fold change in expression) for the intestine samples of the two groups. If the TPM value of the gene was lower than 1, the gene was considered to be not expressed in the intestines of the group. **(C)** Volcano plots of the DEGs for intestines of the 2 groups. The up-regulated genes are presented by red dots, and the down-regulated genes are presented by green dots. **(D)** The functional classification of DEGs for intestines of the 2 groups based on first-category KEGG pathway analysis. **(E)** The functional classification of DEGs for intestines of the 2 groups based on second-category KEGG pathway analysis. **(F)** Heatmap of DEGs for intestines of the 2 groups in the category of “cell growth and death”. Veh: vehicle-treated group; CORT: corticosterone-treated group.

To determine the transcriptional changes in the small intestines after CORT administration, we profiled the transcriptomics of small intestines by RNA-seq. The PCA (principal-component analysis) is shown in **Figure 3A**. After identifying the DEGs (genes with > 1.5-fold change in expression, p < 0.05), we found that there were 1155 genes with significant differences between the two groups (**Figure 3B**). Of the 1155 DEGs, 628 genes were up-regulated and 527 genes were down-regulated (**Figure 3C**). We further examined the functional roles of these genes. By KEGG analysis, 33 DEGs in the category of “Cell growth and death” were identified (**Figure 3D**). Enrichment analysis showed that the 33 DEGs were enriched in the pathways of Cell cycle, Apoptosis and Pathway in cancer (**Figure 3E**). The heatmap indicated that of the 33 genes, 11 genes were up-regulated and 22 genes were down-regulated in CORT-treated group. We identified 5 genes (*Glul*, *Wee1*, *Xiap*, *Casp1* and *Casp7*) (**Figure 3F**), which were reported to mediate apoptosis resistance and cell proliferation (13–22). This result suggested that CORT-induced increased intestinal length might be due to activation of signaling pathways associated with cell proliferation and apoptosis resistance.

### 2.4 Identification of DEGs in response to the CORT treatment in liver

**Figure 4:**
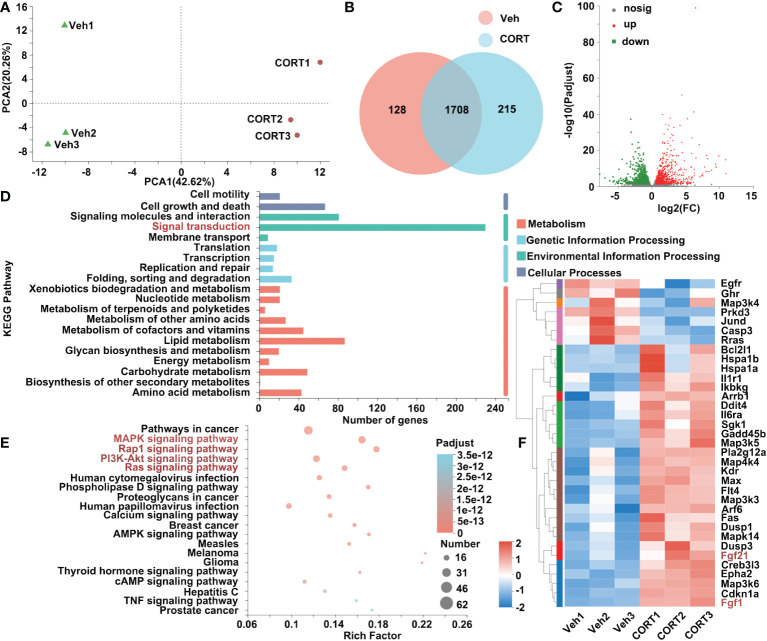
Identification of genes that were differentially expressed in livers in response to CORT treatment. **(A)** Principal component analysis of the transcriptome of the liver samples collected from the mice administrated with CORT or Vehicle. **(B)** Venn diagram displaying the distribution of DEGs (genes with > 1.5-fold change in expression) for the liver samples of the two groups. If the TPM value of the gene was lower than 1, the gene was considered to be not expressed in the livers of the group. **(C)** Volcano plots of the DEGs for the liver samples of the two groups. The up-regulated genes are presented by red dots, and the down-regulated genes are presented by green dots. **(D)** The functional classification of DEGs for livers of the 2 groups based on first-category KEGG pathway analysis. **(E)** The functional classification of DEGs for livers of the 2 groups based on second-category KEGG pathway analysis. **(F)** Heatmap of DEGs commonly expressed in the 4 pathways (labeled in red, 4E) for livers of the 2 groups in the category of “signal transduction”. Veh: vehicle-treated group; CORT: corticosterone-treated group.

To better understand the impact of CORT, we also investigated the effects of CORT on the liver, the key organ for metabolism. After CORT treatment, the profile of transcriptomics changed dramatically. The PCA of RNA-seq is shown in **Figure 4A**. After identifying the DEGs (genes with > 1.5-fold change in expression, p < 0.05), we found that there were 2051 genes with significant differences between the CORT-treated and the control groups (**Figure 4B**). We further examined the functional roles of the 2051 genes. Of the 2051 DEGs, 1132 genes were up-regulated and 919 genes were down-regulated (**Figure 4C**). Among these genes, more than 220 genes are classified into Signal transduction (**Figure 4D**). Gene enrichment analysis showed signal transduction pathways (including MAPK, Rap1, PI3K-Akt and Ras signaling pathways) significantly changed in the liver when treated with CORT (**Figure 4E**). As shown in **Figure 4F**, 35 genes belonging to these pathways were significantly changed. Importantly, both fibroblast growth factor 1 (*Fgf1*) and *fibroblast growth factor 21* (*Ffg21*), which could trigger these pathways, were increased after CORT treatment (**Figure 4F**). The increased expression levels of *Fgf1* and *Ffg21* in mice livers were also confirmed by quantitative real-time PCR (qPCR) (**Figure S1A**). This result indicated that the CORT-induced increased intestinal length and nutrients absorptive capability might be mediated by FGF signaling network, which functions to regulate cell growth and maintain the function and morphology of gastrointestinal tract.

## 3 Discussion

The present study showed: (1) CORT treatment led to longer small intestines, which was then proved to be positively correlated to weight gain; (2) The CORT-treated mice displayed longer intestinal villi with enhanced proliferation and reduced apoptosis of intestinal cells; (3) RNA-seq analysis of the small intestines and the livers, indicating possible signaling pathways involved in the CORT-induced obesity.

Obesity is likely to be caused by calorie surpluses, in which energy intake initially exceeds energy expenditure (10). Accumulated evidence suggests that the increase of GCs plays an important role in the development of obesity (2). CORT-treated mice became obese, even when the food intake was restricted to the same amount as consumed by the control mice (6). This prompted us to raise a question whether CORT-treatment could cause higher absorptive capacity and improved nutrient absorption, thus leading to obesity or fat accumulation. In the present study, the CORT-treated mice exhibited obese phenotype (**Figures 1C–G**), longer small intestines (**Figures 1H, I**) and elongated intestinal villi (**Figures 2A, B**), which are reported to contribute greatly to the enormous absorptive surface area (7–9). Indeed, the unique features for the small intestines, such as circular folds and villi, can increase the absorptive surface area of the small intestine more than 600-fold, thus leading to more efficient nutrient absorption and obesity. In this study, male but not female mice were used as the experimental subjects. Inclusion of female mice in the scientific study is a controversial issue due to the fact that female mice go through an estrous cycle, during which their hormone concentrations change in a 4-to-5 day schedule (23). And most of the scientists argued that the response of female mice might vary too much to be used in the experiments if they are at different time points of their estrous cycle. Actually, accumulated evidence suggested that sex hormones could affect metabolism and distribution of adipose tissues and even obesity (24). Therefore, female mice were not included in the present study due to the reason that sex hormone might become a key variable and add undesirable noise for data collection and analysis.

The positive relationship between body weight and the intestinal length (**Figure 1K**) indicated that obesity might be attributed to CORT-induced intestinal cell proliferation and enormous absorptive surface area. However, we could not exclude the possibility that CORT-induced the central nervous system (CNS)-mediated hyperphagia might also cause obesity. Indeed, the feeding behavior is regulated by CNS to integrate peripheral hunger and satiety signals, subsequently regulating the energy balance and weight status. Feeding behavior is often distinguished by the separation of homeostatic and hedonic processes. Even in the presence of sufficient energy storage, the hedonic drive may override homeostatic control by enhancing the desire to consume delicious meals. Key CNS regions involved in the hedonic pathway include dopaminergic reward centers (nucleus accumbens, amygdala, ventral tegmental area) and the prefrontal impulsivity-control networks (25). In the central nucleus of the amygdala, long-term GC treatment stimulates the production of corticotropin-releasing factor (CRF). CRF enables the recruitment of a chronic stress network, which alters the normal output of autonomic, neuroendocrine and behavioral systems, leading to an increased HPA axis activity. Increased activation of this system leads to an increase in the consumption of high-energy foods (26), which in turn causes increased adipose tissue storage and even metabolism-associated disorders.

Consistent with the well-established role of CORT as a crucial regulator for glucose metabolism-associated hormones, such as leptin and insulin (27–29), the present study exposed that chronic CORT-treated mice showed remarkably increased levels for serum leptin and insulin (**Figures 1L, M**). This result is consistent with a previous study, which reported the elevated plasma leptin and insulin in chronic CORT-treated mice (30). In clinical studies: Newcomer et al. reported that short-term GCs treatment increased the plasma leptin concentration in a dose- and time-dependent manner (31); Plasma leptin levels in patients with Cushing’s syndrome were significantly higher than those in nonobese healthy participants and obese subjects without any metabolic or endocrine illnesses, indicating the role of GCs as a key regulator for leptin synthesis and secretion (32). The controversy remains about how GCs affect leptin secretion. One clinical study indicates that chronic exposure to endogenous GCs leads to insulin resistance, and GCs-induced hyperinsulinemia may act as a mediator of the steroid-induced elevation in leptin levels (33). However, a number of scientists suggest that the steroid-induced rise in leptin levels are not fully dependent on the hyperinsulinemia caused by GCs. According to the study from Veldman et al., patients with Cushing’s disease produced about twice as much leptin in an amplitude-specific manner, and this increased leptin concentration cannot be attributed to insulin production, indicating a cortisol-dependent change in adipose leptin secretion (34). Indeed, *in vitro* studies conducted on rodent adipocytes have demonstrated that GCs increased the mRNA level and secretion of leptin. These effects seem to be mediated by the direct transcriptional actions of GCs on glucocorticoid responsive elements (GRE) present in the leptin promoter (35).

Intestinal surface expansion depends on division of intestinal stem cells in the intestinal crypts and apoptosis of intestinal epithelial cell. Then we asked whether GCs could affect the proliferation or apoptosis of intestinal cells to enlarge intestinal surface area. The present study showed that CORT treatment enhanced the proliferation (**Figures 2C–F**) and inhibited the apoptosis of intestinal cells (**Figures 2G–J**). RNA-seq analysis showed that CORT up-regulated the expression of proliferation-related genes (such as Glul, Wee1 and Xiap) and down-regulated the expression of apoptosis-related genes (such as Casp1 and Casp7) (**Figure 3F**).

Glutamine plays a crucial role in maintaining a balance between proliferation and apoptosis of intestinal cells for normal functions (13). The expression level of glutamate-ammonia ligase (Glul, a gene of glutamine synthetase) was significantly up-regulated in CORT-treated small intestines, which is consistent with the previous studies which suggested that CORT could induce Glul expression in leukemia cells (14) and also in normal tissues (15). Although the function of CORT-induced Glul expression remained unclear, a previous study reported that inhibition of Glul decreased the proliferation of intestinal epithelial cells *in vitro* (16). Therefore, we speculated that CORT-induced Glul expression in mice small intestines might partly contribute to intestinal proliferation. High expression of Wee1 is positively corelated with cell proliferation and malignancy (17); and Wee1 inhibition could suppress gastrointestinal stromal tumor cell proliferation, and induce apoptosis and cell cycle arrest (18). Caspase family are essential for the initiation and execution of apoptosis (20). In murine model of intestinal ischemia-reperfusion (ICR) injury, the expression levels of Casp1 and Casp7 were significantly increased, while reducing Casp1 and Casp7 suppressed ICR-induced intestinal epithelial cell apoptosis (21, 22). Xiap showed its apoptosis resistance effect by repressing Casp7 using both an active site-directed mechanism and non-competitive mechanism (19).

All the blood leaving the stomach and intestines passes through the liver, which is a critical hub for numerous physiological processes, including macronutrient metabolism, lipid and cholesterol homeostasis, and the breakdown of xenobiotic drugs. As shown in **Figure 4**, RNA-seq analysis indicated the up-regulated *Fgf1* and *Fgf21* in CORT-treated livers. FGF signaling network plays a pivotal role in maintaining the function and morphology of gastrointestinal tract, such as WNT/FGF-dependent epithelial proliferation, FGF/RAS-MARK-mediated cell growth and differentiation, and development and repair processes of the gastrointestinal tract (36, 37). Dignass et al. showed that FGF1 (also known as acidic FGF, aFGF) enhanced epithelial cell proliferation (investigated on IEC-6, Caco-2, Caco-29 cell lines) (38). Interestingly, a mutant form of FGF1, which lost mitogenic activity, was proved to be more efficient at reducing cell apoptosis in the gut epithelium of rats following ICR injury (39). In addition, FGF1-treatment could attenuate ICR injury-induced excessive apoptosis in intestinal epithelium by promoting the proliferation of intestinal crypt cells (40, 41). Although the role of up-regulated *Fgf21* in CORT-treated livers is largely unknown, several studies suggested that FGF21 might play a crucial role in maintaining the functions and morphology of intestines. Although FGF21 is primarily produced in the liver, a previous study reported a novel role of FGF21 as a hormonal factor contributing to neonatal intestinal function (42): the *Fgf21*-knockout (KO) pups fed with *Fgf21*-KO milk (lack of FGF21) showed a mild decreased body weight, whereas *Fgf21*-KO pups fed with WT milk (containing FGF21) showed up-regulated intestinal peptides, digestive enzymes, lactase enzymatic activity and normalized galactose levels and body weight. In addition, Sodium butyrate-treated mice showed increased proliferation of intestinal crypt cells, increased length of intestines and increased level of FGF21 in the liver, which might indicate the role of FGF21 in regulating intestinal cell proliferation (43). Further study should be carried out to explore the role of FGF21 in regulating intestinal function and morphology.

Obesity is a documented side effect of long-term GCs exposure, and GCs exert their physiological and pharmacological effect by binding to the nuclear receptor-glucocorticoid receptor (GR). A growing body of evidence suggests that GR responses to GCs to function as a regulator for adipose metabolism, including adipogenesis and lipolysis (44–46). Point-mutation (47) and polymorphisms (48–50) in GR gene are reported to be associated with obesity. We noticed the CORT-treated mice showed obesity and significantly decreased expression level of *Nr3c1*, a gene encodes GR, in the livers (**Figure S1B**). Consistent with the present study, a previous study reported that mice administered with CORT (4 weeks, drinking CORT in water) showed increased body weight and food intake, but decreased expression level for *Nr3c1* in hypothalamus (51). According to clinical studies, long-term GC therapy usually causes the repression of *Nr3c1* gene expression, which in turn causes a reduction in GC sensitivity and subsequently the development of acquired GC resistance (52). However, the precise factor controlling this process remains elusive. Indeed, the mechanisms for controlling *Nr3c1* expression and translation are intricate and tightly regulated in tissues. Up to 13 variants have been identified in the untranslated exon 1 of human *Nr3c1*, functioning to regulate the transcription, the translation and the stability of mRNAs (53). In the present study, long-term CORT exposure-induced *Nr3c1* down-regulation might be mediated by a negative feedback loop: one of the key factors affecting how cells respond to GCs is the GR expression level (54). Through a mechanism known as homologous down-regulation, GCs induce a decrease in GR levels in the majority of cells (55). As a result, the range of cellular responsiveness to GCs is constrained by this ligand-induced, receptor-mediated process. However, we could not exclude the possibility that alterations in GR expression/activation in brain or other tissues might also contribute to chronic CORT exposure-induced obesity. Further studies by genetic manipulation of GR expression in the interested organs of experimental animals might help to uncover the role of GR in CORT-induced obesity.

Above all, chronic CORT-treatment promotes intestine proliferation and inhibits apoptosis for intestinal cells *via* multiple metabolism-associated signaling pathways, thus leading to enlarged intestine-mediated more nutrient absorption and obesity. Understanding these altered metabolism-associated pathways might provide novel therapeutic clues for GCs or stress-associated obesity.

## 4 Materials and methods

### 4.1 Housing of animals and CORT-treatment experiment

Male C57BL6/J mice (age: 8 weeks; weight: 23-25 g, n=32) purchased from Charles River Laboratories (Beijing, China) were maintained in a Specific Pathogen Free animal facility with standard conditions (room temperature: 24°C; humidity: 55%) under a 12:12 light/dark cycle. Food and water were provided ad libitum. All interventions and animal care procedures have been approved by the Animal Committee of School of Life Sciences and Technology, Tongji University, Shanghai, China.

Two groups of the weight-matched mice were injected with different solutions: either CORT or Vehicle. For drug trials, 34.7 mg corticosterone (Topscience, T0948L) was dissolved in 1ml DMSO, then 18.8 mL sterile water containing 200 μL Tween-80 was added into the solution to reach a final concentration of 1.734 mg/mL. Mice were administered with CORT (40 mg/kg) or vehicle (23.07 mL/kg) every day *via* subcutaneous (s.c.) injection for 8 weeks. Food intake (once a week), water intake (twice a week) and body weight (once a week) were recorded.

### 4.2 Body composition analysis by MRI

After 8 weeks of CORT injection, the mice were anesthetized and the body composition of mice was evaluated using a small animal *in vivo* MRI system (NM21-060H-I, Suzhou Niumag Analytical Instrument Corporation, Suzhou, China), according to the manufacturer’s instructions. Body composition was evaluated by T1-weighted images, and body fat was evaluated using the Meso NM21-060H-I sober small animal body composition analysis and imaging system (magnetic field strength, 0.5 T; Repetition Time (TR)/Echo Time (TE) = 360 ms/18.2 ms; matrix: 128 * 128; thickness: 3 mm).

### 4.3 Preparation of frozen tissue sections and histological investigation

At the end of the study, the whole-body perfusion and fixation was performed once the animal is under general anesthesia. Then the livers and intestines were isolated and transferred into 30% sucrose for dehydration. Then the completely dehydrated tissues were embedded in O.C.T. compound. Then freeze and store the embedded tissues at -80°C freezer until sectioning. The frozen tissue blocks were sectioned at 10-μm thickness using a cryostat (Lecia CM 1950), then the sections were stained for histological investigation using Hematoxylin-eosin (H&E) staining kit (Sangon Biotech, E607318-0200) and Oil Red O staining kit (Sangon Biotech, E607319-0010), following the manufacturer’s instructions. H&E image of swiss-rolled intestine was divided into 4 quadrants and 10 intact villi from each quadrant were randomly selected for measurement. Villus length was then determined as the distance from the distal edge of the crypt base to the villus apex.

### 4.4 *In vivo* proliferation assay and immunofluorescence staining

*In vivo* proliferation assay was performed as previously described elsewhere (43) with some modifications. To visualize proliferative cells, mice were intraperitoneally injected with 5 mg/kg of EdU dissolved in sterile PBS and euthanized 24 hours later. Intestinal tissues were Swiss-rolled and dehydrated. Then the frozen sections were prepared as described above. Sections for intestinal rolls were stained for EdU using the Click-iT Alexa Flour 488 Imaging Kit (Thermo Fisher Scientific, C10337). The length from crypt to the EdU-labelled cells furthest from the crypt were measured from 40 villi in the duodenums of each mouse using ImageJ software.

Immunofluorescence staining was performed as we previously described (56) with some modifications. Slides were washed twice in PBS and incubated in blocking buffer (10% donkey/goat serum in PBST) for 1 hour at room temperature (RT). Then the slides were incubated with primary antibodies diluted in incubation buffer (5% donkey/goat serum in PBST) overnight at 4°C. After washing for 3 times, the slides were then incubated with Biotin-SP AffiniPure Goat Anti-Rabbit lgG (H+L) secondary antibody (Yesen, 33103ES60, dilution 1:200) for 2 hours at RT. Slides were then washed and incubated with Alexa Fluor 488 Streptavidin (Yesen, 35103ES60, dilution 1:400) and DAPI (Yesen, 40727ES10, dilution 1:1000). After washing with DPBS, the slides were mounted with anti-fade fluorescence mounting medium (Southern Biotech, 0100-01, Birmingham, USA). Imaging was performed using Olympus IX73 microscope system. Primary antibodies used for immunofluorescence: Ki67 (rabbit, Abcam ab16667, dilution 1:200), Cleaved Caspase 3 (CC3) (rabbit, Cell Signaling Technologies 9579S, dilution 1:200). The crypt depth was determined by Ki67-labeled cells to show the depth of the invagination between 2 villi. Deeper villi crypts indicate a higher metabolism state, allowing for the renewal and proliferation of the enterocytes. Immunofluorescence staining of CC3 was used to indicate enterocyte apoptosis. The number of CC3+ cells in each villus was counted using ImageJ software.

### 4.5 TUNEL assay

Terminal deoxynucleotidyl transferase dUTP nick end labeling (TUNEL) assay for detecting DNA fragments (apoptosis) was performed on frozen sections of duodenum using Click-IT TUNEL AlexaFluor647 kit (Roche, Cat. No. 11684795910), following the manufacturer’s instructions. TUNEL assay was used to indicate enterocyte apoptosis. The number of TUNEL+ cells in each villus was counted using ImageJ software.

### 4.6 Transcriptional analysis by RNA-sequencing

The liver and intestine tissues (duodenum) of experimental mice were isolated and stored at -80°C until use. Then the frozen tissues (50 mg/sample) were send for RNA-extraction and RNA-seq analysis (Majorbio Bio-pharm Technology Co.,Ltd, Shanghai, China). Briefly, total RNA was extracted from different groups with three replicates. RNA-seq transcriptome library was prepared with 1 μg of total RNA using TruSeqTM RNA sample preparation Kit from Illumina (San Diego, CA). Then the paired-end RNA-seq library was sequenced with the Illumina HiSeq xten/NovaSeq 6000 sequencer (2 × 150 bp read length). The raw paired-end reads were trimmed and quality was controlled by SeqPrep (https://github.com/jstjohn/SeqPrep) and Sickle (https://github.com/najoshi/sickle), followed by aligning to the reference genome with orientation mode using HISAT2 software (57). The mapped reads of each sample were assembled by StringTie in a reference-based approach (58). The expression level of each transcript was calculated according to the transcripts per million reads (TPM) method. The TPM value of each DEG was shown in **supplementary files**. Transcript abundances were quantified using RSEM software tool (59). DEGs analysis was performed by DESeq2 with adjusted P value ≤ 0.05. KEGG pathway analysis were performed using KOBAS (http://kobas.cbi.pku.edu.cn/home.do).

### 4.7 mRNA extraction and qPCR

The tissues were promptly isolated, placed into TRIzol and homogenized in a homogenizer (-50°C, 65 Hz) for 60 s. The residual genomic DNA was removed by DNase I treatment. The total RNA was diluted in RNAase free water, then 1 μg of RNA was reverse transcribed to cDNA using PrimeScriptTMRT reagent kit (TAKARA). qPCR was performed using the SYBR Green Mix (Bio-Rad) in a BioRad CFX96 Thermal Cycler. Housekeeping gene beta-actin was used to normalize mRNA levels between different samples. Primer sequences are listed as follows:

*Fgf1*-forward: GAGGATCCTTCCTGATGGC
*Fgf1*-reverse: CTCATTTGGTGTCTGCGAG
*Fgf21*-forward: GTCATTCAAATCCTGGGTGTC
*Fgf21*-reverse: AAAGTGAGGCGATCCATAGAG
*Nr3c1*-forward: AGAGGACAACCTGACTTCC
*Nr3c1*-reverse: ATTCCAGGGCTTGAATATCCA
*beta-actin*-forward: GCTATGTTGCTCTAGACTTCG
*beta -actin*-reverse: GGATTCCATACCCAAGAAGG

### 4.8 Blood collection from orbital sinus and serum measurement by enzyme-linked immunosorbent assay

The experimental mice were anesthetized, and the skin around eye was pulled away to allow the eye to bulge out of the socket. Then a sterile capillary tube was penetrated into the corner of the eye socket underneath the eyeball to collect blood from orbital sinus into an Eppendorf tube. The samples were centrifuged (4°C, 3000 rpm, 15 min), then the supernatants were collected into Eppendorf tubes and stored at -80°C until use. The concentrations of leptin, insulin and triglycerides were detected by using the ELISA kits (leptin: KE10048, Proteintech; Insulin: ab277390, Abcam; Triglyceride: MAK266, Sigma-Aldrich.), according to the manufacturer’s instructions.

### 4.9 Statistical analysis

Statistical analysis was performed using GraphPad PRISM 8 (GraphPad Software, San Diego, CA, USA). Unpaired student’s t-test was used to compare the difference between two groups. Simple linear regression was used to analyze correlations. Pearson’s R2 coefficient, with confidence interval 95% and two-tailed P value were calculated to test the effectiveness of pairing. All data were expressed as the mean ± the standard error of the mean (SEM). *p < 0.05, **p < 0.01, ****p < 0.0001 were considered to be significant.

## Data availability statement

The data presented in the study are deposited in the Gene Expression Omnibus (GEO) repository, accession number GSE214009.

## Ethics statement

The animal study was reviewed and approved by Animal Committee of School of Life Sciences and Technology, Tongji University, Shanghai, China.

## Author contributions

GW planned and performed molecular and animal experiments, analyzed data, produced the figures and wrote the manuscript. SL, YL, MZ and TX performed molecular experiments and collected the data. TL performed animal experiments. LC produced the figures, wrote the manuscript and provided financial support. JL designed and conceived this project, provided financial support, wrote the manuscript and approved the manuscript. All authors contributed to the article and approved the submitted version.
