# Supplementary material for: Corticosterone induces obesity partly via promoting intestinal cell proliferation and survival
Source: Front Endocrinol (Lausanne). 2023 Jan 9;13:1052487. doi: 10.3389/fendo.2022.1052487 (PMC9869250; doi:10.3389/fendo.2022.1052487)
Supplement: Supplementary file 7 [file DataSheet_3.pdf]

**Supplementary Figures**

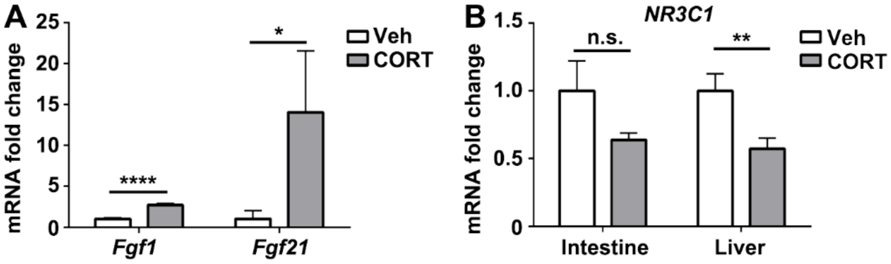


**Supplementary Figure 1. (A)** Evaluation of the mRNA expression levels for *Fgf1* and *Fgf21* in the livers from Vehicle- or CORT-treated mice. **(B)** Evaluation of the mRNA expression level for *NR3C1* in intestines or livers from the two groups of mice. Data are expressed as mean ± SEM, *p < 0.05, **p < 0.01, ****p < 0.0001, n.s.: no significance.
