# Supplementary figures and images for: Corticosterone induces obesity partly via promoting intestinal cell proliferation and survival
Source: Front Endocrinol (Lausanne). 2023 Jan 9;13:1052487. doi: 10.3389/fendo.2022.1052487 (PMC9869250; doi:10.3389/fendo.2022.1052487)

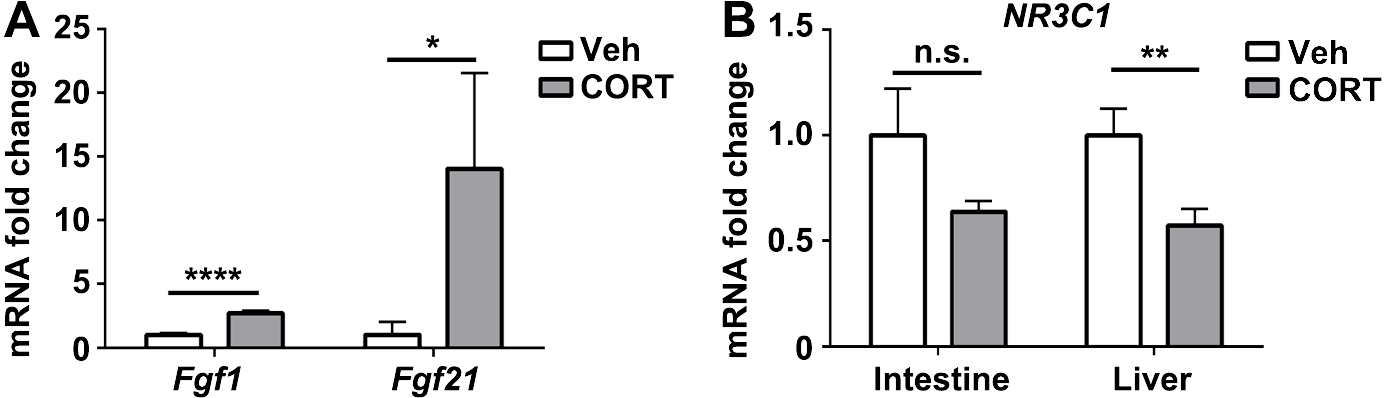

Supplement: Supplementary file 8 [file Image_1.tif]
